# Autologous cell-free serum preparations in the management of knee osteoarthritis: what is the current clinical evidence?

**DOI:** 10.1186/s43019-020-00036-5

**Published:** 2020-03-23

**Authors:** Darshan S. Angadi, Hamish Macdonald, Navraj Atwal

**Affiliations:** Department of Trauma and Orthopaedics, Cheltenham General Hospital, Sandford Rd, Cheltenham, UK

**Keywords:** Autologous, Protein, Solution, Conditioned, Serum, Osteoarthritis, Knee

## Abstract

**Background:**

There is paucity in the current literature regarding clinical outcomes of autologous cell-free serum preparations. The objective of this paper is to collate the clinical evidence and review the results of intraarticular injections of autologous cell-free serum preparations in the management of knee osteoarthritis (OA).

**Methods:**

A comprehensive English literature search was undertaken using the healthcare database website (https://hdas.nice.org.uk/). The PubMed, Medline, CINAHL, Embase and the Cochrane library databases were searched to identify all studies of autologous protein solution/autologous conditioned serum (ACS/APS) in the management of knee OA. We evaluated the reported clinical outcomes with respect to pain, function, morbidity, adverse effects and complications.

**Results:**

Fifteen relevant articles were identified in the current literature. Outcomes following injection of ACS/APS have been reported in patients with age range (34–87 years) and unilateral or bilateral knee OA. Seven studies reported improvement in visual analog scale (VAS) whereas the Western Ontario and McMaster Universities osteoarthritis instrument (WOMAC) score improved in nine studies. Considerable variation was noted in the injection technique and duration of post-procedure assessment with only one study reporting long-term follow-up beyond 24 months. Joint swelling and injection-site pain were reported to be the most common complications; only one study reported a case of septic arthritis. However, no evidence is available to clearly identify factors that may predict the outcomes following this procedure.

**Conclusion:**

Current data from the clinical studies would suggest that the intraarticular administration of autologous cell-free serum preparations, such as ACS/APS, in patients with knee OA may improve pain and function, with limited morbidity. High-quality clinical trials with stratified patient cohorts, longer follow-up duration and robust reporting of outcome measures are essential to improve the understanding of the indications and clinical effectiveness of these novel products.

## Background

Osteoarthritis (OA) of the knee is common [1], debilitating [2] and increasing in its prevalence [1]. Any therapeutic intervention that can relieve symptoms of OA, prevent its progression and/or delay the need for surgery therefore has potential to dramatically improve quality of life for patients. Nonoperative treatment modalities for symptomatic knee OA include analgesia [3], physiotherapy [4], healthy lifestyle and weight reduction regimens [5]. Surgical intervention in the form of osteotomy [6] or arthroplasty [7] is well-established, but is not without complications [8] and a prolonged rehabilitation phase [9].

Intraarticular therapies for knee OA using intraarticular corticosteroid (IAC) and hyaluronic acid (HA) have been reported [10]. However, dose-dependent chondrotoxicity [11], short duration of effect [12] with IAC and arthralgia [13], and variable evidence [14] with HA are some of their limitations. Some investigators have performed intraarticular injection of platelet-rich plasma (PRP) [15] and mesenchymal stem/stromal cells (MSCs) [16] due to their perceived biological function(s) to restore joint homeostasis [12, 17]. The pathophysiology of OA includes a complex interplay of pro-inflammatory mediators like interleukin-1 (IL-1), interleukin-6 (IL-6), tumor necrosis factor-alpha (TNF-a), macrophage chemotactic protein (MCP), monokine induced by interferon (MIG), oncostatin M (OSM) and matrix metalloproteinases (MMPs) amongst others. The biological therapeutic group products which have been developed for the management of OA of the knee are designed to inhibit the action of interleukin-1 (IL-1), a pro-inflammatory cytokine that has been implicated in, and targeted for treatment of, multiple human diseases [12, 17, 18]. Animal experimental evidence suggests that upregulation of IL-1-receptor antagonist (IL-1Ra) may reduce the progression of OA [19]. Biological injective therapy options based around this theory have been developed and referred to as autologous conditioned serum (ACS) or autologous protein solution (APS), both of which are prepared from autologous peripheral blood, which is conditioned by incubation with glass beads, leading to increases in the production of IL-1Ra as well as multiple other cytokines and growth factors [17].

Several investigators have evaluated the role of autologous cell-free serum preparations like APS [20–22] and ACS [23–25] in the management of knee OA with human clinical studies following initial in-vitro [18, 26] and animal model studies [19, 27]. The majority of the available review articles on the topic have summarised the basic science and the results of ACS. However, there is paucity in the current literature providing an objective evaluation of the clinical outcomes of these novel products.

Hence, the primary objective of this paper was to collate the available clinical evidence in the published literature and critically appraise the results of intraarticular injection of autologous cell-free serum preparations in the management of knee OA. The secondary objective was to answer the following questions encountered in the clinical decision-making process of managing patients with OA of the knee:

1. Is it safe to perform intraarticular injection of autologous cell-free serum preparations such as APS/ACS for OA of the knee using commercially available kits?
2. Does this procedure provide an effective and long-lasting symptomatic relief to avoid further surgical intervention?
3. What factors (patient/procedure/device) significantly influence the outcome following this treatment?
4. Do these intraarticular injection procedures have any potential adverse effect on the outcomes of subsequent surgical procedures?

Given the similarities, we included studies pertaining to both, with the generic title of ACS/APS referring to both products.

## Methods

### Literature search and databases

An English literature search of all the available evidence was undertaken (June 2019) using the healthcare database website (https://hdas.nice.org.uk/). The databases searched were Medline, CINAHL, Embase and the Cochrane library.

### Search criteria

Medline search was performed using Boolean statements and the wildcard symbol (*). The search criteria: “knee* AND (auto* OR autologous*) AND (condition* OR conditioned* OR protein* OR pro*) AND (serum* OR solution* OR solutions*)”. Embase search was performed using Boolean statements and the wildcard symbol (*). The search criteria: “knee* AND (auto* OR autologous*) AND (condition* OR conditioned* OR protein* OR pro*) AND (serum* OR solution* OR solutions*)”. CINAHL database was searched using the following criteria: “knee* AND (auto* OR autologous*) AND (condition* OR conditioned* OR protein* OR pro*) AND (serum* OR solution* OR solutions*)”.

A review of the Cochrane database for relevant articles was performed. An adjunctive bibliography search was undertaken to identify additional relevant studies through review articles and Google scholar (https://scholar.google.co.uk/) using the commercial names of APS –‘nSTRIDE’(Biomet Biologics, Warsaw, IN, USA) and ACS –‘Orthokin’(Orthogen AG, Dusseldorf, Germany). Additionally, given the scope of the current review to assess the safety of this novel treatment including adverse reactions, a comprehensive search of the grey literature (OpenGrey [28]/OpenDOAR [29]) was undertaken. All studies reporting the clinical outcomes of patients with knee OA receiving intraarticular injection of autologous cell-free preparations like APS/ACS were included. Review articles, animal and in-vitro studies were excluded.

## Results

The above database search returned 555 articles of which 27 were relevant to the current review. Adjunctive bibliography search identified three articles whereas 15 articles were noted to be duplicates (Table 1). Grey literature search found no results. Thus, a total of 15 relevant articles were identified in the current literature and were selected for further review.

**Table 1:** Results of literature search

	Database				Total
	Medline	Embase	CINAHL	Cochrane	
**Search results**	132	362	50	11	555
**Relevant articles**	8	11	5	3	27
**Adjunctive bibliography**					03
**Duplicates**					− 15
**Studies for review**					15

It was noted that amongst the 15 articles, two were initial abstracts [25, 30] of subsequent papers in the same cohort of patients. Hence, data from the later studies was considered for analysis. Details of the articles which describe the clinical outcomes of intraarticular injection of autologous cell-free serum preparations like APS/ACS are provided in Tables 2, 3, 4 and 5. The salient points and current evidence to the focussed questions to help in the clinical decision-making process are presented below.

**Table 2:** List of relevant studies from literature

Author and year	Study patients	Age at presentation Mean (± SD)/median (range)	BMI Mean (± SD)/median (range)	OA severity (K-L grade/ACR criteria)	Intraarticular injection and number	Follow-up (months) Mean (± SD)/median (range)	Outcome measures	Adverse events (AE)/complications	Level of evidence
Kon et al. 2018 [22]	46	57 (41–68)	NR	K-L 2–3	APS 1	12 (± NR)	WOMAC VAS KOOS SF-36 CGI-S/PGI-S	AE – 6 Arthralgia	2
Tassara et al. 2018 [24]	25	68 (34–87)	NR	NR	ACS 4	6 (± NR)	VAS ROM	None	3
Zarringam et al. 2018 [23]	126	63 (NR)	NR	K-L 1–3	ACS 6	90 (± 47)	K-M	NR	3
Barreto et al. 2017 [31, 32]	100	61.2 (± 1.2)	33.8 (± 1.4)	ACR	ACS 6	12 (± NR)	VAS XSMFA-D PGIC	NR	3
Hang et al. 2017 [33]	92	NR	NR	NR	ACS 4	12 (± NR)	VAS WOMAC	NR	3
Shirokova et al. 2017 [34]	123	59.9 (± 8.8)	NR	ACR	ACS 6	3 (± NR)	VAS WOMAC	NR	3
Hix et al. 2017 [20]	10	58.8 (± 9.5)	29.0 (± 3.9)	NR	APS 1	12 (± NR)	WOMAC KOOS NRS	AE – 4 Arthralgia Discomfort	3
van Drumpt et al. 2016 [21]	10	57.5 (± 9.5)	26.6 (± 3.1)	K-L 1–4	APS 1	18 (± 1)	WOMAC	AE – 6 Arthralgia Joint stiffness Injection site pain	3
Garcia-Escudero et al. 2015 [35]	118	59 (34–81)	29.6 (± NR)	K-L 1–4	ACS 4	24 (± NR)	WOMAC NRS	None	3
Rutgers et al. 2015 [36]	20	50 (34–70)	NR	K-L 1–3	ACS 6	12 (± NR)	VAS KOOS KSCRS	NR	3
Motaal et al. 2014 [37]	30	54.21 (± 5.95)	NR	K-L 1–3	ACS 3	3 (± NR)	WOMAC	NR	3
Baltzer et al. 2009 [38]	376	53.8 (± 12.2)	NR	K-L 2–3	ACS 6	25 (± NR)	WOMAC SF-8 VAS	AE – 31 Joint swelling Transient pain	2
Yang et al. 2008 [39]	153	54 (± 11)	27 (± 5)	K-L 1–3	ACS 6	12 (± NR)	WOMAC KSCRS	Knee pain – 44 Irritation – 70 Swelling – 10 Septic arthritis – 1	2

*AE* adverse events*, BMI* body mass index, *ROM* range of movement, *SD* standard deviation, *NR* not reported, *APS* autologous protein solution, *ACS* autologous conditioned serum, *K-L* Kellgren-Lawrence, *ACR* American College of Rheumatology criteria, *VAS* visual analogue scale, *KOOS* Knee injury and Osteoarthritis Outcome Score, *WOMAC* Western Ontario and McMaster Universities osteoarthritis instrument, *OA* osteoarthritis, *SF-8* Short-Form 8 health-related quality of life, *K-M* Kaplan-Meier method, *CGI-S* Clinical Global Impression of Severity, *PGI-S* Patient Global Impression of Severity, *XSMFA-D* Extra Short Musculoskeletal Functional Assessment, *KSCRS* Knee Society clinical rating scale, *NR*

**Table 3:** Reported outcomes – visual analogue scale (VAS) following intraarticular injection of autologous conditioned serum/autologous protein solution (ACS/APS)

Author and year	Study patients	Age at presentation Mean (± SD)/median (range)	Follow-up (months) Mean (± SD)/median (range)	Outcome measure	Baseline	Post procedure	
					Score Mean (± SD)/median (range)	Time point(s) (weeks/months)	Score Mean (± SD)/median (range)
Kon et al. 2018 [22]	46	57 (41–68)	12 (± NR)	VAS	5.5 (± NR)	12 months	NR^a^
Tassara et al. 2018 [24]	25	68 (34–87)	6 (± NR)	VAS	80 (70–100)	1 month	20 (0–60)
						6 months	20 (0–60)
Barreto et al. 2017 [31, 32]	100	61.2 (± 1.2)	12 (± NR)	VAS	5.8 (± 0.6)	1 week	4.2 (± NR)
						2 weeks	3.3 (± NR)
						3 months	3.1 (± NR)
						6 months	3.2 (± NR)
						12 months	2.3 (± NR)
Hang et al. 2017	92	NR	12 (± NR)	VAS	6.07 (± NR)	12 months	2.52 (± NR)
Shirokova et al. *2*017 [34]	123	59.9 (± 8.8)	3 (± NR)	VAS	NR^b^	3 months	NR^b^
Rutgers et al. 2015 [36]	20	50 (34–70)	12 (± NR)	VAS	52.20 (± 22.90)	12 months	50.05 (± 23.76)
Baltzer et al. 2009 [38]	376	53.8 (± 12.2)	25 (± NR)	VAS	69.6 (± 13.10)	6 months	29.5 (± 22.58)

*NR*

^a^visual analogue scale (VAS) at 12 months reported to be 49% better but numeric score not provided

^b^VAS at 3 months reported to be 47% better but VAS value not provided

**Table 4:** Reported outcomes – Western Ontario and McMaster Universities osteoarthritis instrument (WOMAC) following intraarticular injection of autologous conditioned serum/autologous protein solution (ACS/APS)

Author and year	Study patients	Age at presentation Mean (± SD)/median (range)	Follow-up (months) Mean (± SD)/median (range)	Outcome measure	Baseline	Post procedure	
					Score Mean (± SD)/median (range)	Time point(s) (weeks/months)	Score Mean (± SD)/median (range)
Kon et al. 2018 [22]	46	57 (41–68)	12 (± NR)	WOMAC	51.2 (± NR)	12 months	NR^a^
Hang et al. 2017	92	NR	12 (± NR)	WOMAC	Function – 70.3 (± NR)	12 months	Function – 27.2 (± NR)
					Mobility – 8.6 (± NR)	12 months	Mobility – 3.1 (± NR)
					Pain – 20.9 (± NR)	12 months	Pain – 9.0 (± NR)
Shirokova et al. 2017 [34]	123	59.9 (± 8.8)	3 (± NR)	WOMAC	NR^b^	3 months	NR^b^
Hix et al. 2017 [20]	10	58.8 (± 9.5)	12 (± NR)	WOMAC	Pain – 12.0 (± 1.2)	12 months	Pain – 3.3 (± 2.9)
van Drumpt et al. 2016 [21]	10	57.5 (± 9.5)	18 (± 1)	WOMAC	55 (± NR)	12 months	20 (± NR)
Garcia-Escudero et al. 2015 [35]	118	59 (34–81)	24 (± NR)	WOMAC	Global – 81.6 (± NR)	12 months	Global – 35.2 (± NR)
					Function – 60.4 (± NR)	12 months	Function – 29.4 (± NR)
					Stiffness – 3.36 (± NR)	12 months	Stiffness – 3.3 (± NR)
					Pain – 17.9 (± NR)	12 months	Pain – 2.5 (± NR)
Motaal et al. 2014 [37]	30	54.21 (± 5.95)	3 (± NR)	WOMAC	Total – 45.63 (± 9.99)	1 week	Total – 26.23 (± 11.50)
						1 month	Total – 8.0 (± 7.28)
						2 months	Total – 7.52 (± 6.91)
						3 months	Total – 8.27 (± 5.45)
Baltzer et al. 2009 [38]	376	53.8 (± 12.2)	25 (± NR)	WOMAC	Global – 5.24 (± 2.32)	7 weeks	Global – 2.80 (± 2.30)
						13 weeks	Global – 2.42 (± 2.06)
						26 weeks	Global – 2.42 (± 2.19)
					Pain – 5.18 (± 2.39)	7 weeks	Pain – 2.71 (± 2.37)
						13 weeks	Pain – 2.33 (± 2.14)
						26 weeks	Pain – 2.42 (± 2.25)
					Stiffness – 5.59 (± 2.7)	7 weeks	Stiffness – 3.07 (± 2.49)
						13 weeks	Stiffness – 2.80 (± 2.33)
						26 weeks	Stiffness – 2.78 (± 2.45)
					Function – 5.21 (± 2.41)	7 weeks	Function – 2.80 (± 2.34)
						13 weeks	Function – 2.40 (± 2.08)
						26 weeks	Function – 2.37 (± 2.21)
Yang et al. 2008 [39]	153	54 (± 11)	12 (± NR)	WOMAC	54.49 (± 17.6)	3 months	63.37 (± 20.6)
						6 months	62.90 (± 23.7)
						9 months	61.78 (± 23.4)
						12 months	65.02 (± 24.1)

*NR*

^a^WOMAC at 12 months reported to be 49% better but numeric score not provided

^b^WOMAC at 3 months reported to be 28.7% better but numeric score not provided

**Table 5:** Reported outcomes – Knee injury and Osteoarthritis Outcome Score (KOOS) and numerical rating score (NRS) following intraarticular injection of autologous protein solution/autologous conditioned serum (ACS/APS)

Author and year	Study patients	Age at presentation Mean (± SD)/median (range)	Follow-up (months) Mean (± SD)/median (range)	Outcome measure	Baseline	Post procedure	
					Score Mean (± SD)/median (range)	Time point(s) (weeks/months)	Score Mean (± SD)/median (range)
Kon et al. 2018 [22]	46	57 (41–68)	12 (± NR)	KOOS	39.9 (± NR)	12 months	NR
Hix et al. 2017 [20]	10	58.8 (± 9.5)	12 (± NR)	KOOS	36.9 (± 16.2)	12 months	79.7 (± 16.2)
Rutgers et al*.* 2015 [36]	20	50 (34–70)	12 (± NR)	KOOS	49.45 (± 11.46)	12 months	51.20 (± 13.09)
Hix et al. 2017 [20]	10	58.8 (± 9.5)	12 (± NR)	NRS	5.9 (± 1.9)	12 months	1.6 (± 1.6)
Garcia-Escudero et al. 2015 [35]	118	59 (34–81)	24 (± NR)	NRS	8.10 (± NR)	12 months	3.03 (± NR)

*NR* Not reported

### Safety profile and morbidity of intraarticular injections of ACS/APS

To date, the outcomes of 1229 patients involved in studies evaluating the effect of injection of ACS/APS have been reported (Table 2). The adverse reactions noted in these patients have been similar to the intraarticular administration of other therapeutic agents [17, 40]. Additionally, they have been of a transient and self-limiting type [12, 41]. Following clinical trials, the investigators from the above studies in the current literature have concluded that the overall safety profile of autologous cell-free serum preparations, such as ACS and APS, to be satisfactory for clinical use. Ongoing clinical trials [42] have the scope to provide further evidence in this aspect. Amongst the various studies in the literature only one study from 2008 reported a single case of septic arthritis [39]. However, this complication has not been reported by other investigators.

### Duration of symptom relief following ACS/APS injection

Current studies have demonstrated that duration of pain and symptom relief period can vary from 3 to 24 months (Tables 2 and 4). Zarringam et al*.* [23] performed Kaplan-Meier survivorship analysis on the original cohort of patients from the study conducted by Yang et al. [39] between February 2004 to August 2006. They noted that at 7.5 ± 3.9 years of follow-up, 40.3% of patients from the group who received ACS compared to 46.3% of patients from the placebo group who received physiological saline underwent surgical intervention. However, this was not statistically significant (*p* = 0.150).

### Factors influencing the outcome of ACS/APS treatment

#### Patient demographics and physiology

Outcomes following injection of ACS/APS have been reported in patients over a wide age range (34–87 years) with unilateral or bilateral knee OA. All the investigational studies of these products have been performed on cohorts of patients with symptomatic knee OA excluding patients with conditions like systemic/inflammatory joint disease and crystalline/neuropathic arthropathy. It must be noted that whilst some investigators excluded patients who had had surgery on their knee within 3 months [31, 38] of the screening visit others have extended this time limit to 6 [22] and 12 months [21]. Furthermore, there is significant variation in the inclusion criteria with respect to patients who have received intraarticular treatments. Body mass index (BMI) of the study patients is not consistently reported in the literature (Table 2).

#### Indications and severity of OA

ACS/APS injections have been performed in patients with symptomatic unilateral or bilateral knee OA with radiographic changes (Kellgren-Lawrence grade 1 to 4). However, there is no clear agreement in terms of the objective criteria used to assess the severity of knee OA prior to performing these injections in the study patients. A combination of American College of Rheumatology (ACR) criteria [31, 38], visual analogue scale (VAS) [38, 39], numeric rating scale (NRS) [35], Western Ontario and McMaster Universities osteoarthritis instrument (WOMAC) [21, 22, 39], including the pain subscale, have been used by the investigators.

#### Mechanical parameters (range of motion/lower limb alignment)

Only one study [24] in the literature has evaluated the effect of the intraarticular administration of ACS on range of motion. Tassara et al. [24] noted that the median range of knee flexion improved by 25° to 120° (95–140°) from a baseline of 95° (90–105°). Currently, there is no data to draw conclusions regarding varus/valgus alignment of the knee and its influence on the outcome following injection of ACS/APS.

#### Injection technique and regimen

Considerable variation is noted with the injection regimen for ACS (Table 2). Some investigators [31, 37] have used 1 ml whilst others [24, 34, 35, 39] have administered 2 ml of ACS. Furthermore, the number of injections performed is variable ranging between three [37] to six [31, 36] over a period of 2–3 weeks. In contrast all the studies [20–22] with APS used a single injection of 2.5 ml. Some authors [21, 23, 39] recommend aspiration of knee prior to injection whilst others have used ultrasound-guided injections [22].

### Implications for future surgical procedures

Five studies [21, 23, 35, 36, 38] have described further procedures carried out on participants subsequent to intraarticular treatment with ACS/APS. Only limited and indirect data is available from the current studies regarding patients who have undergone surgical procedures such as partial/total knee replacement [23, 35] and osteotomy [23]. In patients who had previously received ACS/APS there were a total of 35 additional procedures (26 arthroplasties, four osteotomies, two arthroscopic interventions and three unknown surgeries). To date no adverse outcome has been reported in this sub-group of patients following surgical intervention. One study [38] stated that 122 patients received further interventions, including surgical, pharmacological and complementary therapies, but did not give further information or stratify these by the therapy previously provided (ACS, HA or saline placebo). None of the trials specifically examined complications following subsequent procedures.

## Discussion

### Basic science of ACS/APS

#### Production methods

Currently, several commercially available kits are utilised to prepare ACS/APS from whole-blood samples of patients [32, 38, 43]. In general, both ACS and APS are prepared from autologous peripheral blood, the volume of which can vary between 10 and 60 ml depending on the manufacturer kit [20, 37, 38]. The obtained blood sample is further conditioned by incubation with glass beads and centrifugation, leading to an increase in the production of IL-1Ra as well as multiple other cytokines and growth factors [17, 20].

#### Composition

The predominant cytokine in ACS is IL-1Ra with an average concentration of 2015 compared to 236 picograms per milliliter (pg/ml) in a basal blood sample [41]. Additional components of ACS described include platelet-derived growth factor (PDGF), transforming growth factor-beta (TGF-β), insulin-like growth factor (IGF), and fibroblast growth factor (FGF) amongst others [41, 44, 45]. Hix et al. [20] reported the average concentration of IL-1Ra in APS to be 63,740 pg/ml whereas Woodell-May et al*.* [46] noted it to be 30,853 pg/ml. The wide variation in the IL-1Ra levels of ACS and APS reported by the investigators may be due a combination of the production kit and the 24-h incubation period used during the preparation of ACS [47]. Various anabolic factors, including epidermal growth factor (EGF), vascular endothelial growth factor (VEGF), IGF, TGF-β and the anti-inflammatory cytokines soluble tumor necrosis factor receptor type II (sTNF-RII), IL-4 and IL-10, have been noted in APS [46].

#### Potential mechanisms of action in OA

Broadly, both ACS and APS are injectable solutions enriched in endogenous cytokines which help to restore joint homeostasis preventing degenerative changes in cartilage and bone [17, 21, 32, 44, 48]. They offer a non-surgical intraarticular treatment plan at the molecular level which incorporates the inhibition of IL-1b through the rapid induction of IL-1Ra.

It has been demonstrated that following interaction with medical-grade concentrator beads, the post-conditioned serum level of IL-1Ra is significantly elevated in relation to IL-1b [20, 49]. This relative increase alters the relative ratio of IL-1Ra to IL-1b that is essential to restore homeostasis of joints affected by OA [50].

Whilst IL-1Ra is a major component of these novel agents, their biochemical constituents contain various combinations of PRP and several growth factors present in the a granules of platelets [51]. The aforementioned growth factors, which include TGF-β, PDGF, VEGF and IGF, have been shown to stimulate chondrocyte proliferation and augment articular cartilage metabolism [44, 52]. Whilst it is perceived that both ACS and APS potentially mitigate the inflammatory cascade, the detailed pathways or mechanisms through which they perform these actions have not been fully described [40, 45, 47, 53].

### Limited follow-up and natural history of OA

There is paucity of robust data in the literature to evaluate the long-term benefits of autologous cell-free serum preparations in the management of knee OA. This is vital for a condition such as OA with multifactorial aetiology and treatments that influence the prognosis. Zarringam [23] et al. attempted to answer this question in the cohort of patients who had participated in a study [39] a decade earlier. It must be observed that in this study the composition of patient groups that reported the outcomes were based on certain assumptions thereby limiting the interpretation of the results.

### Imaging features

In their study Kon et al*.* [22] analysed the effect of the intraarticular administration of APS on the size of bone-marrow lesion and osteophytes over a 12-month period using the Magnetic resonance imaging OsteoArthritis Knee Score (MOAKS). They observed that in the central zone of the lateral femoral condyle these lesions significantly improved in the study population. However, these changes were not statistically significant. Of note, neither the study protocol as listed on ClinicalTrials.gov [54], nor the ‘Methods’ section, specify how the magnetic resonance imaging (MRI) images were planned to be analysed. Nonetheless, this an area for further investigation as it has been demonstrated that bone-marrow lesions contribute to significant symptoms in patients with knee OA [55, 56].

### Outcome measures and clinical factors

The visual analogue scale (VAS) and Western Ontario and McMaster Universities osteoarthritis instrument (WOMAC) are the most commonly used outcome measures in the current literature (Tables 3 and 4). Some studies have used the Knee Injury and Osteoarthritis Outcome Score (KOOS) and the numeric rating scale (NRS) to report the results of injection of ACS/APS (Table 5). However, there is lack of consensus on the outcome measures to report the results of this procedure.

Some authors [57] have hypothesised that the preparation and administration of autologous cell-free serum preparations in patients with a raised C-reactive protein (CRP) can yield suboptimal results due to the elevated levels of pro-inflammatory cytokines (IL-1, TNF) in the same blood.

There is considerable variation in the technique of intraarticular administration of ACS/APS as highlighted earlier. Some authors [21, 23, 39] recommend aspiration of the knee prior to injection to reduce the risk of drug dilution. However, this step is not reported consistently in the other studies [31, 35, 37]. Synovial fluid analysis is performed as a diagnostic investigation for knee conditions including crystal arthropathy [58]. It is interesting to note that no study has analysed the aspirate fluid to determine the suitability of patients to this treatment procedure.

BMI has been reported to be an independent risk factor for the development [59] and progression of symptoms [60] in patients with knee OA. Limb malalignment in the coronal/sagittal plane can contribute to knee-joint degeneration and wear [61, 62]. However, the current studies lack uniform data on these vital factors which predict the outcome of any intervention in this set of patients (Table 2). Another confounding factor in the studies investigating the effect of ACS/APS is the inconsistent criteria for the use of type and dose of adjunctive analgesics in the study patients. This has to be considered whilst interpreting the results of the studies.

It must be mentioned that the largest series (over 1000 patients) on the topic is available in the German literature [63] and is not included in the current review. Nonetheless, in this study no significant adverse outcomes were reported.

### Cost of ACS/APS treatment regimen

No study in the literature has directly compared the cost of autologous cell-free serum preparations in the management of knee OA with other treatment options. However, it must be mentioned that the cost implications of products like ACS/APS can be inferred from various commercial sources. In their study, Barreto et al. [31] stated that ACS was a relatively cost-effective treatment compared to other injection treatments like PRP, stem cells or surgical procedures like total knee replacement. Orthokin® (Orthogen AG, Dusseldorf, Germany) has a stated cost of €150–750 for ACS therapy (≈ US$170–855) [64], whilst the nSTRIDE® APS kit is quoted at £770 (≈ US$1016) excluding the adjunctive equipment [65]. Synvisc® (Sanofi, Paris, France), a representative HA injection costs £68.33 (≈ US$87.85) per injection [66]. By comparison, Kenalog® (Bristol-Myers Squibb, New York, NY, USA) containing triamcinolone acetonide has a *British National Formulary* indicative price of £1.49 (≈ US$1.92) per 40-mg vial [67]. The cost of PRP therapy varies from £425–1200 (≈ US$545–1540) [68] whereas the guide price for hospital-based autologous stem-cell therapy for knee OA may be higher at £7500 (≈ US$9626) [69].

### Summary

The overall quality of evidence supporting ACS/APS use for OA of the knee is poor, with considerable heterogeneity between trials and a paucity of large, well-conducted randomised controlled trials. There is some evidence that ACS/APS is effective in the short-to-medium term (3–24 months) control of pain from OA as well as improving range of movement and function. The rate of serious complications is low, and there is no evidence that future surgery is compromised by prior ACS/APS injection. There have been no formal health economic analyses of ACS/APS compared to more established intraarticular therapies.

## Conclusions

Limited data from the current studies would suggest that intraarticular administration of autologous cell-free serum preparations, such as ACS/APS, in patients with knee OA may improve pain and function with limited morbidity. Given the heterogenous data in the literature it may be useful to develop blood or synovial tests that may predict an efficacious result from ACS/APS. However, high-quality clinical trials with stratified patient cohorts, longer follow-up duration and robust reporting of outcome measures are essential to improve the current understanding of the indications and clinical effectiveness of these products.

## Data Availability

Presented in the manuscript

## Abbreviations

APS: Autologous protein solution
ACS: Autologous conditioned serum
OA: Osteoarthritis
IAC: Intraarticular corticosteroid
HA: Hyaluronic acid
IL-1: Interleukin-1
IL-1Ra: Interleukin-1-receptor antagonist
IL-6: Interleukin-6
TNF-a: Tumor necrosis factor-alpha
MCP: Macrophage chemotactic protein
MIG: Monokine induced by interferon
OSM: Oncostatin M
MMPs: Matrix metalloproteinases
PRP: Platelet-rich plasma
MSCs: Mesenchymal stem/stromal cells
SD: Standard deviation
NR: Not reported
K-L: Kellgren-Lawrence
ACR: American College of Rheumatology criteria
VAS: Visual analogue scale
KOOS: Knee injury and Osteoarthritis Outcome Score
WOMAC: Western Ontario and McMaster Universities osteoarthritis instrument
SF-8: Short-Form 8 health-related quality of life
K-M: Kaplan-Meier method
CGI-S: Clinical Global Impression of Severity
PGI-S: Patient Global Impression of Severity
XSMFA: D-Extra Short Musculoskeletal Functional Assessment
KSCRS: Knee Society Clinical Rating Scale
BMI: Body mass index
NRS: Numeric rating scale
CRP: C-reactive protein
MOAKS: Magnetic resonance imaging OsteoArthritis Knee Score
